# Supplementary material for: Macrophages migrate persistently and directionally upon entering 2D confinement in the presence of extracellular matrix
Source: Biol Open. 2025 Jul 2;14(7):bio061782. doi: 10.1242/bio.061782 (PMC12264731; doi:10.1242/bio.061782)
Supplement: Supplementary information [file biolopen-14-061782-s1.pdf]

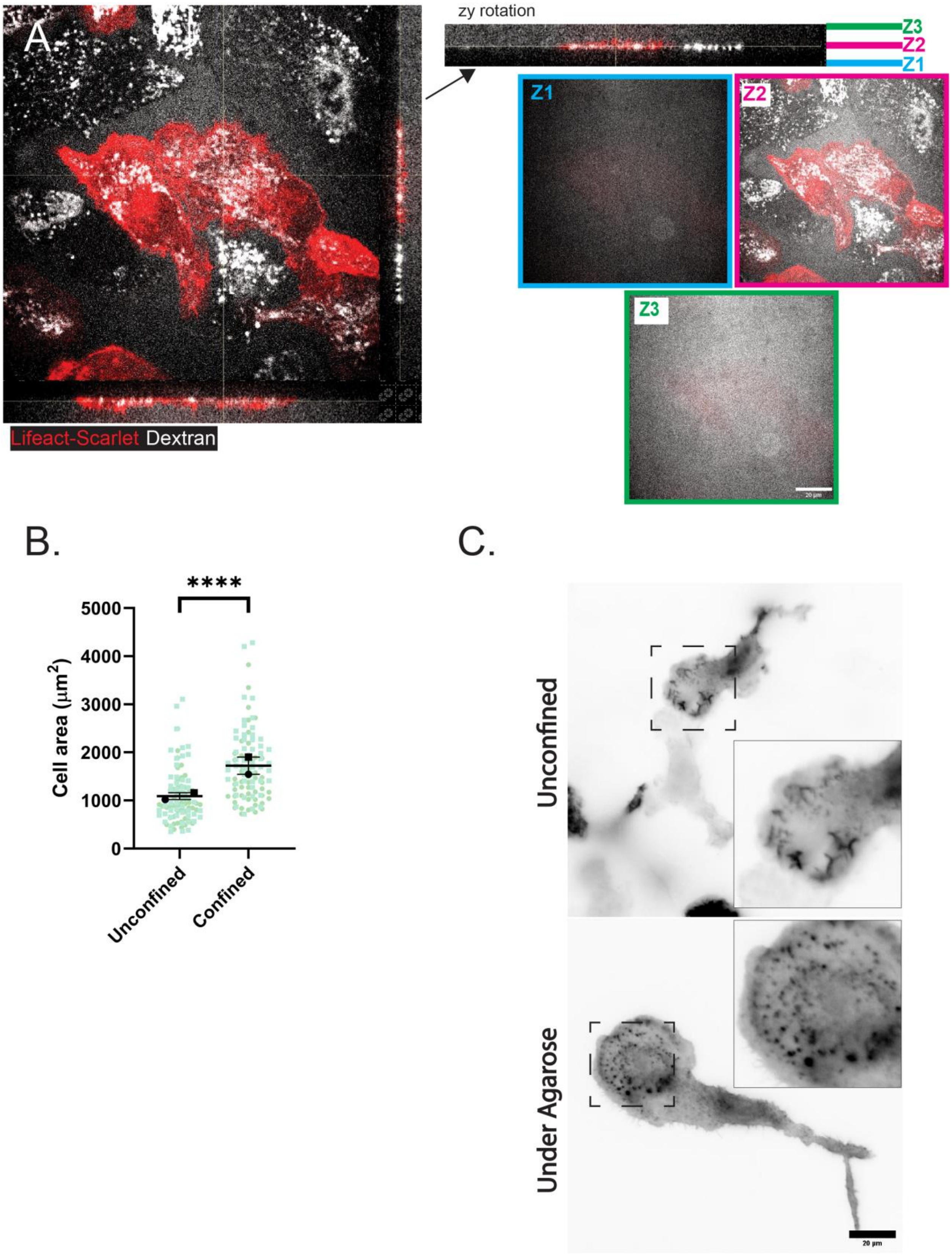

A
zy rotation
Z3
Z2
Z1
Lifeact-Scarlet Dextran
20 µm
B.
****
Cell area (µm2)
5000
4000
3000
2000
1000
0
Unconfined
Confined
C.
Unconfined
Under Agarose
20 µm

Fig. S1. Additional quantification related to Figure 1, part 1. (A) *Left*: Confocal image of Lifeact-Scarlet expressing cells (red staining) within an agarose gel containing fluorescent dextran (white staining). Side projections corresponding to the vertical and horizontal lines across this image are at the right and bottom of this image, respectively. *Right*: The vertical side projection has been rotated (top) and color-coded markers have been included to mark the bottom (Z1), middle (Z2) and top (Z3) of the image series consisting of 43 total images, which is composed of z slices taken at 0.3-micron intervals. Of these, 17 slices span the width of the indicated cell. The images corresponding to the three representative positions (Z1, Z2, Z3) are taken from the 43 total image slices and are reproduced with color-coded borders. As with the image on the left, dextran is colored white and Lifeact-Scarlet is colored red. Scale Bar = 20 microns. (B) Quantification of spread cell area for confined and unconfined cells in the same dish. Experimental means and SEM are represented with black symbols and individual cell area values are plotted for each experiment. Means and values belonging to the same N are shape-coded (circle or square). Statistical analysis was done with Mann-Whitney test. ****$p < 0.0001$. For each condition $n = 100$ cells, pooled from 2 independent experiments. (C) Example images of LA-Scarlet staining in live macrophages in unconfined (top) or agarose-confined (bottom) settings. Scale bar = 20 microns and both images are at the same scale. Insets have been maintained at the same scale relative to each other.

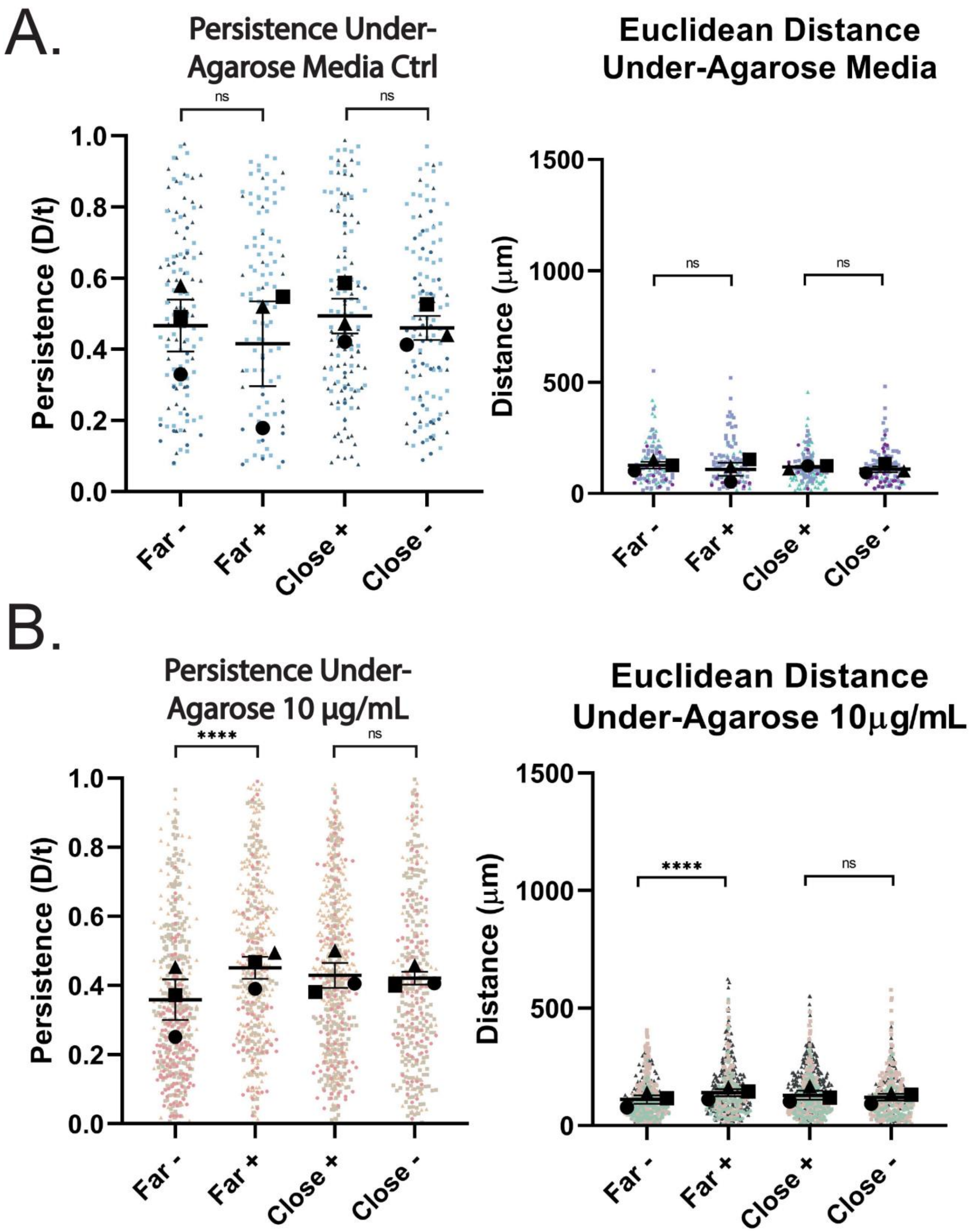

Biology Open • Supplementary information

Fig. S2. Additional quantification related to Figure 1, part 2. (A) Persistence (left) and Euclidean distance (right) of Far +/- and Close +/- macrophages migrating under agarose with media only. Both values were calculated from the same dataset as **Fig. 1C**. Experimental means and SEM are represented with black symbols and all cell migration tracks are plotted and each experimental run is color- and shape-coded (circle, square, or triangle). Statistical analysis was assessed with Kruskal–Wallis and Dunn multiple comparisons test. Ns= not significant. Far- n = 123 tracks, Far+ n = 104 tracks, Close+ n = 139 tracks, Close- n = 123 tracks. These data were pooled from 3 independent experiments. ns = not significant (B) Persistence (left) and Euclidean distance (right) of Far +/- and Close +/- macrophages migrating under agarose in the presence of uniform 10 µg/mL RRX-FN. Both values were calculated from the same dataset as **Fig. 1E**. Experimental means and SEM are represented with black symbols and all cell migration tracks are plotted and each experimental run is color- and shape-coded (circle, square, or triangle). Statistical analysis was assessed with Kruskal–Wallis and Dunn multiple comparisons test. ****$p < 0.0001$, ns = not significant. Far- n = 501 tracks, Far+ n = 460 tracks, Close+ n= 609 tracks, Close- n = 378 tracks. These data were pooled from 3 independent experiments.

Biology Open • Supplementary information

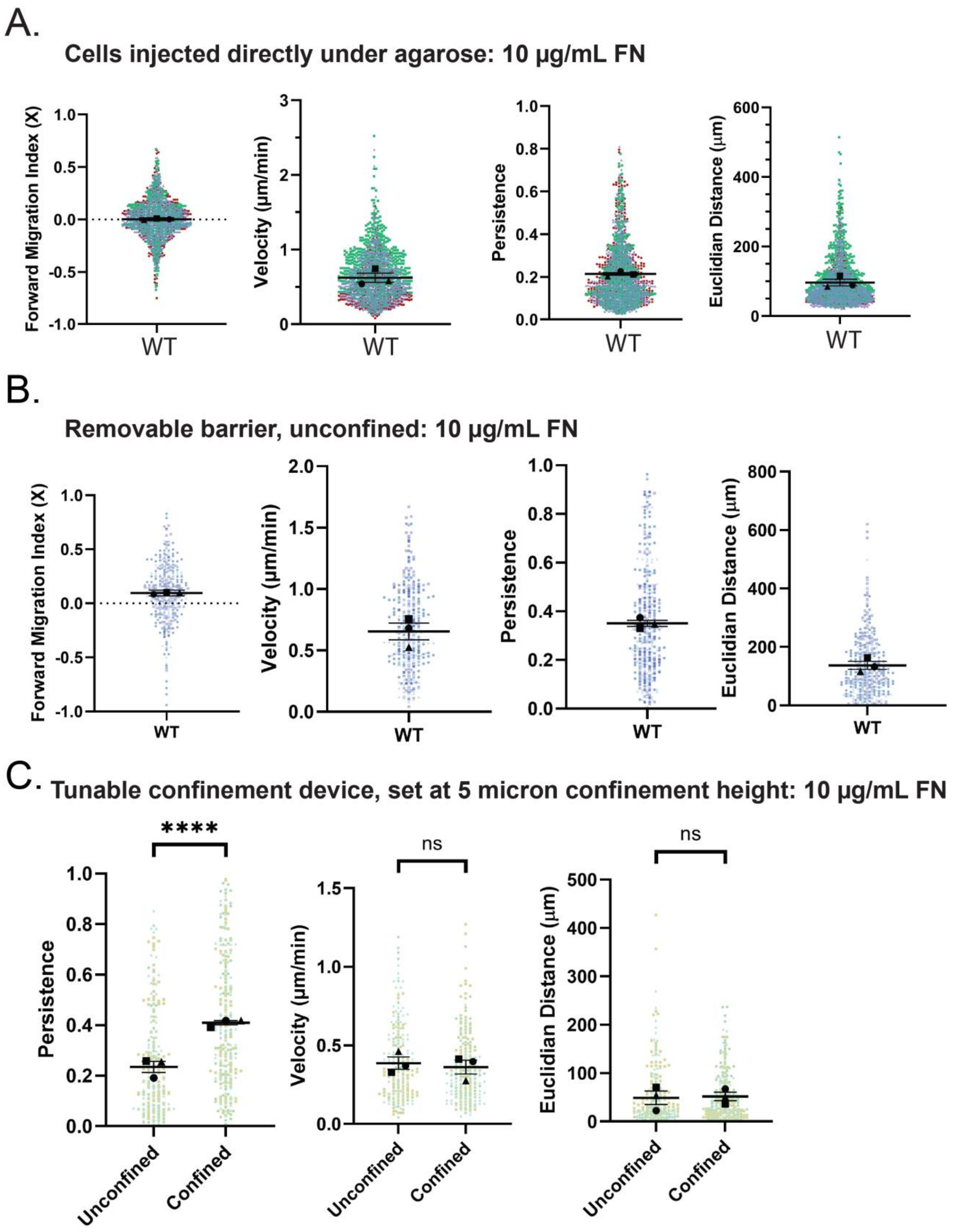
A.
Cells injected directly under agarose: 10 µg/mL FN
Forward Migration Index (X)
Velocity (µm/min)
Persistence
Euclidian Distance (µm)
WT
B.
Removable barrier, unconfined: 10 µg/mL FN
Forward Migration Index (X)
Velocity (µm/min)
Persistence
Euclidian Distance (µm)
WT
C.
Tunable confinement device, set at 5 micron confinement height: 10 µg/mL FN
****
ns
Persistence
Velocity (µm/min)
Euclidian Distance (µm)
Unconfined
Confined

Fig. S3. Migration characteristics of cells injected directly under agarose, contained in a removable barrier, or within a tunable confinement device (all on 10 µg/mL fibronectin). (A) FMIx, velocity, persistence and Euclidean distance of cells injected directly under agarose in presence of uniform 10 µg/mL FN. Experimental means and SEM for each experimental replicate are represented with black symbols, and all individual values are plotted and each experimental run is color- and shape-coded (circle, square, triangle). n = 1,591 tracks. These data were pooled from 3 independent experiments. (B) FMIx, velocity, persistence, and Euclidean distance of cells concentrated in a 0.4 mm well within a removable barrier in the presence of uniform 10 µg/mL fibronectin. All cell migration was quantified after the barrier was removed. Experimental means and SEM for each experimental replicate are represented with black symbols, and all individual values are plotted and each experimental run is color- and shape-coded (circle, square, triangle). n = 376 tracks, pooled from 3 independent experiments. (C) Persistence, velocity and Euclidean distance of confined and unconfined cells in the one well static confinement system, set to a confinement height of 5 microns in the presence of uniform 10 µg/mL fibronectin. All cell migration was quantified immediately after confinement was engaged. Experimental means and SEM for each experimental replicate are represented with black symbols, and all individual values are plotted and each experimental run is color- and shape-coded (circle, square, triangle). Statistical analysis was done by Mann-Whitney test. ****$p < 0.0001$, ns = not significant. n = 248 (unconfined) and 291 (confined) tracks, pooled from 3 independent experiments.

Biology Open • Supplementary information

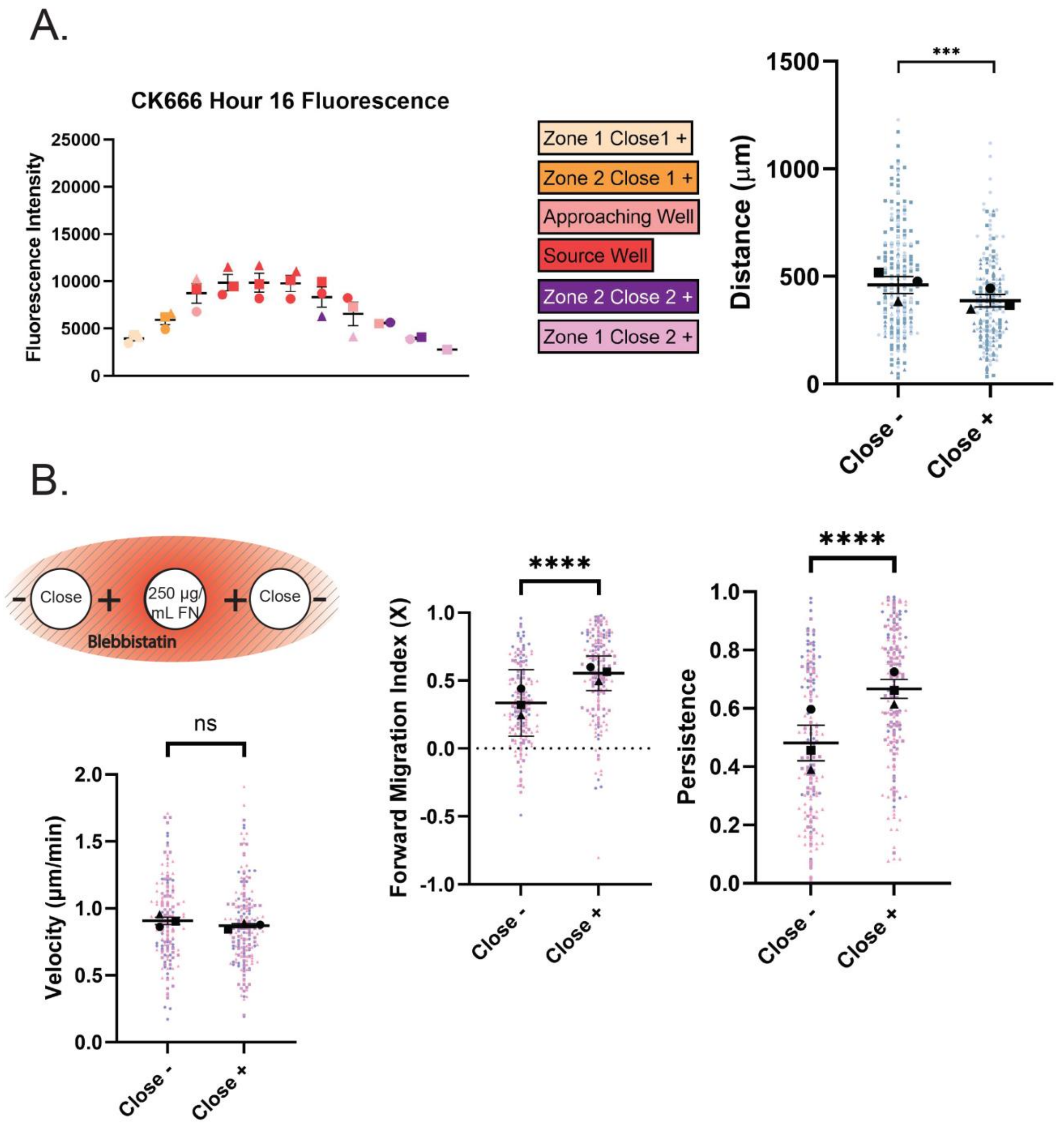

Fig. S4. Additional quantification of CK-666 haptotaxis experiment, and blebbistatin haptotaxis results related to Figure 3. (A) *Left*: RRX-FN fluorescence intensity at the experimental endpoint across the well in CK-666 inhibitor experiments. *Right*: Euclidean distance of Close +/- macrophages migrating under agarose in the presence of an RRX-FN gradient and CK-666. Values were calculated from the same dataset as in Fig. 3B. Means and

SEM are represented with black symbols, and all tracks are plotted and each experimental run is color- and shape-coded (circle, square, or triangle). Statistical analysis was done by Mann-Whitney test. ***p = 0.0002, ns = not significant. Close- *n* = 250 tracks, Close+ *n* = 194 tracks. Data were pooled from 3 independent experiments. (B) *Left*: Schematic depicting Blebbistatin experimental conditions. A 250 µg/mL FN gradient is generated as normal, but in this case the agarose is made up with 30 µM Blebbistatin, and 15 µM Blebbistatin is included in center well, and both cell wells. Solid lines running through the schematic represent Blebbistatin polymerized into the gel. FMIx, persistence and velocity measurements of macrophages moving under agarose in the presence of blebbistatin. Experimental means from each run are represented with 95% confidence interval (FMIx) or SEM (velocity, persistence), and all individual values are color- and shape-coded (circle, square, triangle). Statistical analysis was done by Mann-Whitney test. ****p < 0.0001, ns = not significant. Close- n = 184 tracks, Close+ n = 200 tracks. Data were pooled from 3 independent experiments.

Biology Open • Supplementary information

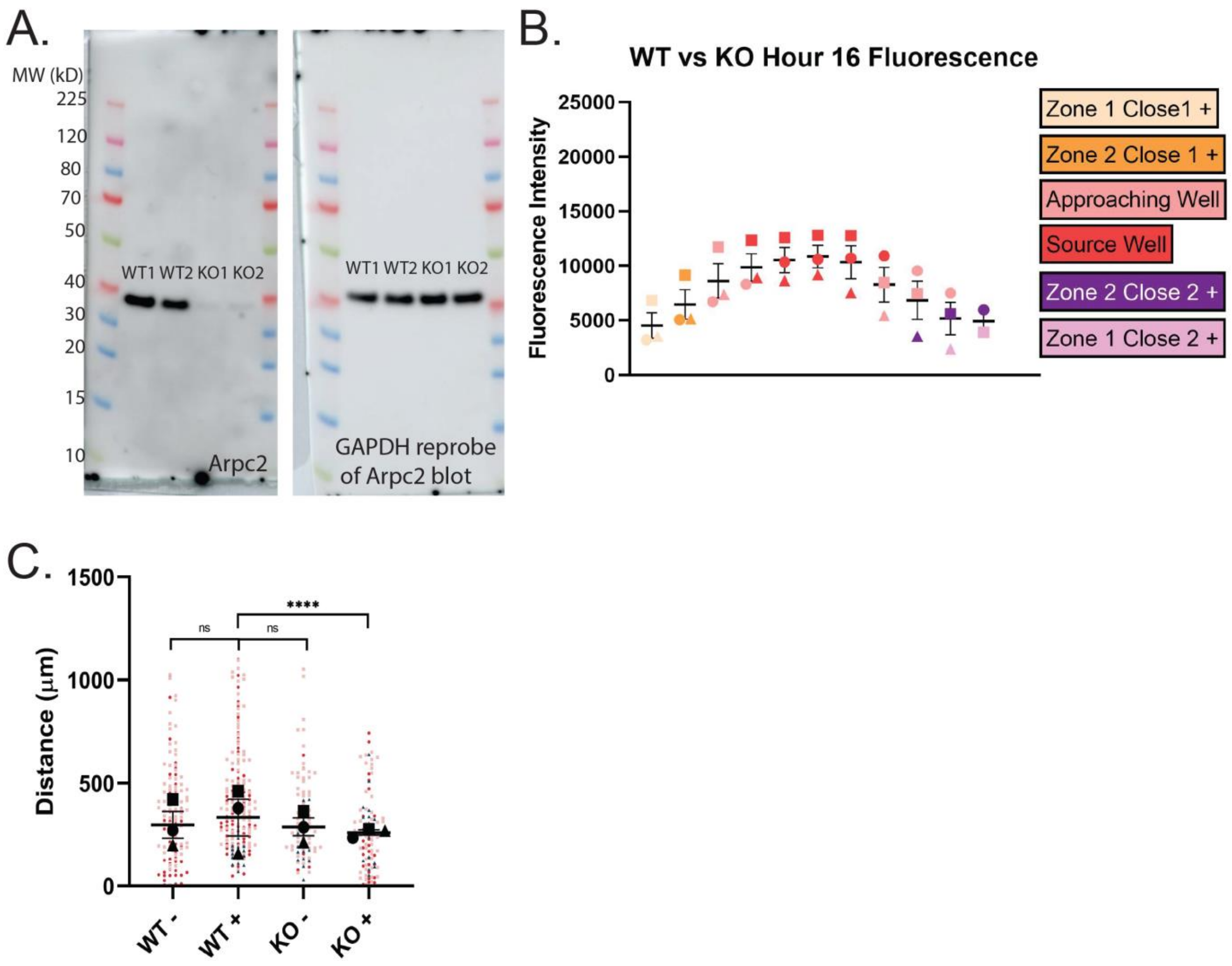

Fig. S5. Validation of *Arpc2-/-* macrophages and additional data from WT and *Arpc2-/-* haptotaxis experiment related to Figure 3. (A) *Left*: Overlay of PVDF membrane and western blot of Arpc2. Two populations left untreated (WT) and two populations treated (KO) with tamoxifen to induce tamoxifen-dependent deletion of the Arpc2 subunit of Arp2/3. *Right*: Re-probe of Arpc2 blot for GAPDH to demonstrate equal loading. Molecular weights of markers are indicated to the left of each blot. (B) RRX-FN fluorescence intensity at the experimental endpoint across the well in WT versus KO haptotaxis experiments. (C) Euclidean distance of WT +/- and *Arpc2-/-* (KO) +/- macrophages migrating under agarose in the presence of an RRX-

FN gradient. Values were calculated from the same dataset as in Fig. 3D. Means and SEM are represented with black symbols, and all cell migration tracks are plotted and each experimental run is color- and shape-coded (circle, square, or triangle). Statistical analysis was done by Kruskal–Wallis with Dunn multiple comparisons test. ****$p < 0.0001$, ns = not significant. WT- $n = 128$ tracks, WT+ $n = 202$, KO- $n = 95$, KO+ $n = 97$. Data were pooled from 3 independent experiments.

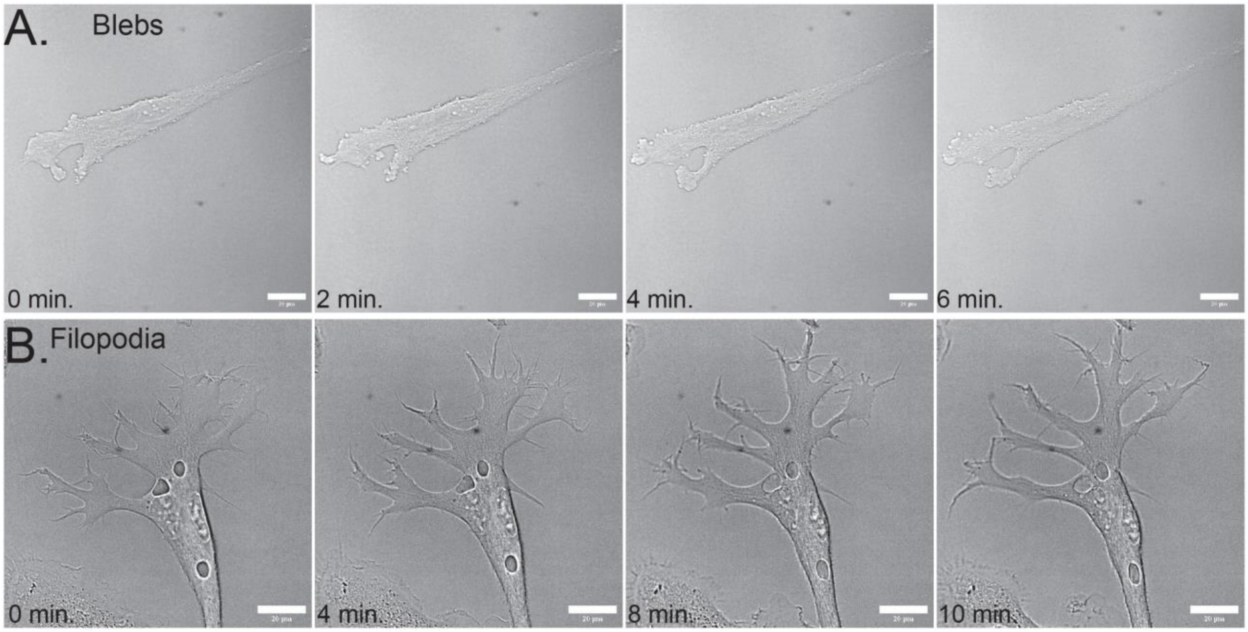

Fig. S6. *Arpc2-/-* protrusion types. (A) Example images of an *Arpc2-/-* macrophage protruding via membrane blebbing. (B) Example images of an *Arpc2-/-* macrophage protruding via filopodial projections. Scale bar = 20 microns

Biology Open • Supplementary information

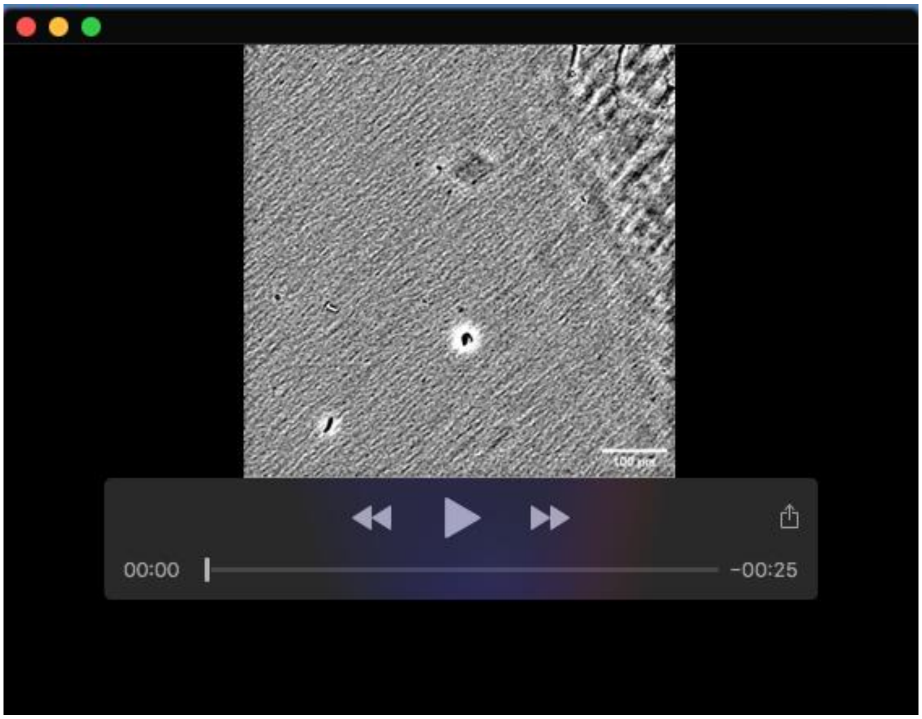

Movie 1. Macrophages in Zone 2 (+) migrating with media only.

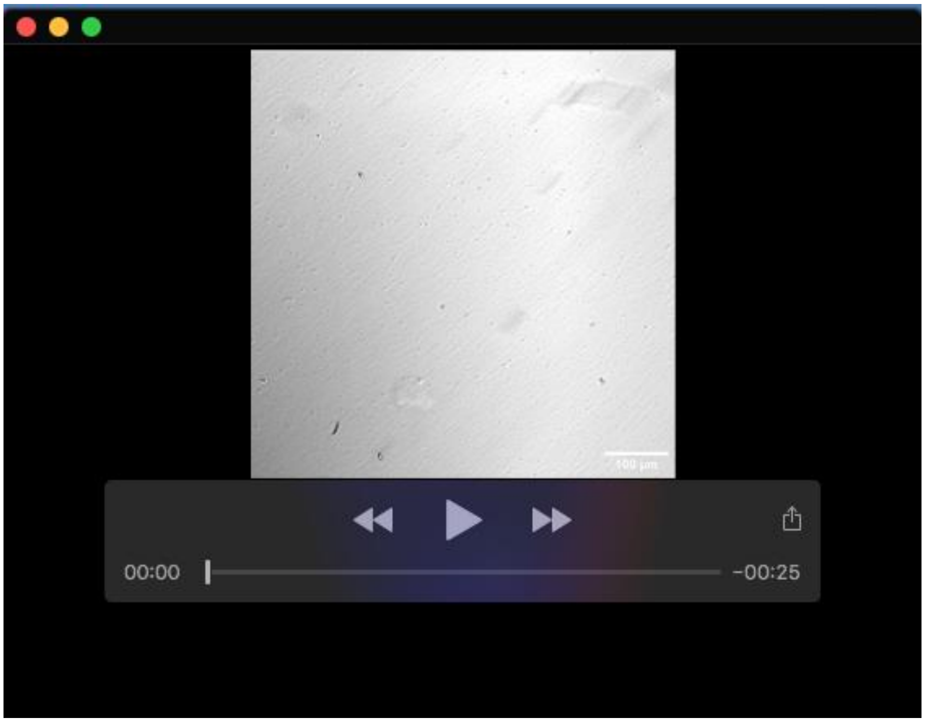

Movie 2. Macrophages in Zone 2 (-) migrating with media only.

Biology Open • Supplementary information

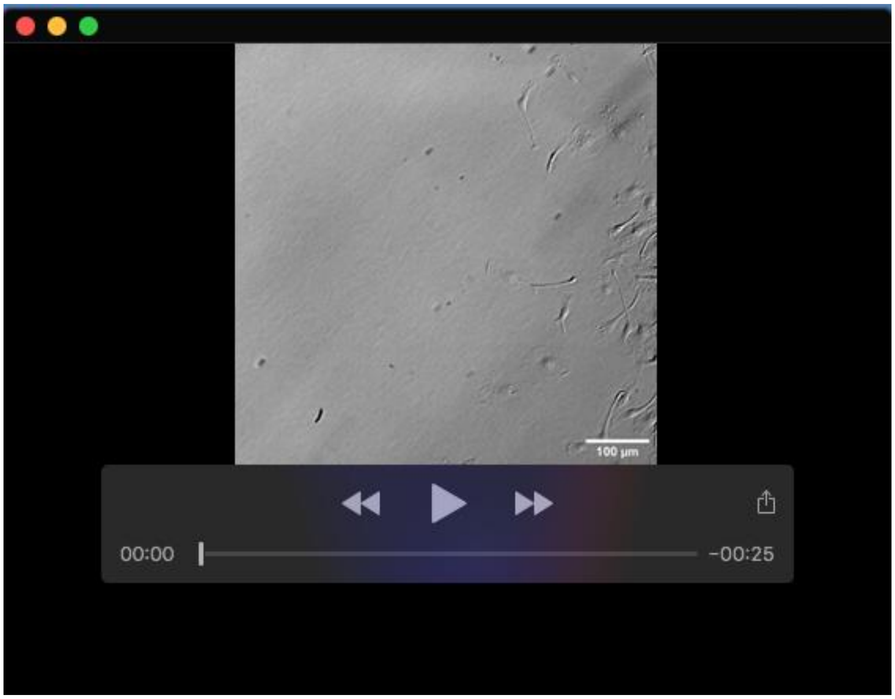

Movie 3.: Macrophages in Zone 2 (+) migrating on 10 ug/mL uniform fibronectin.

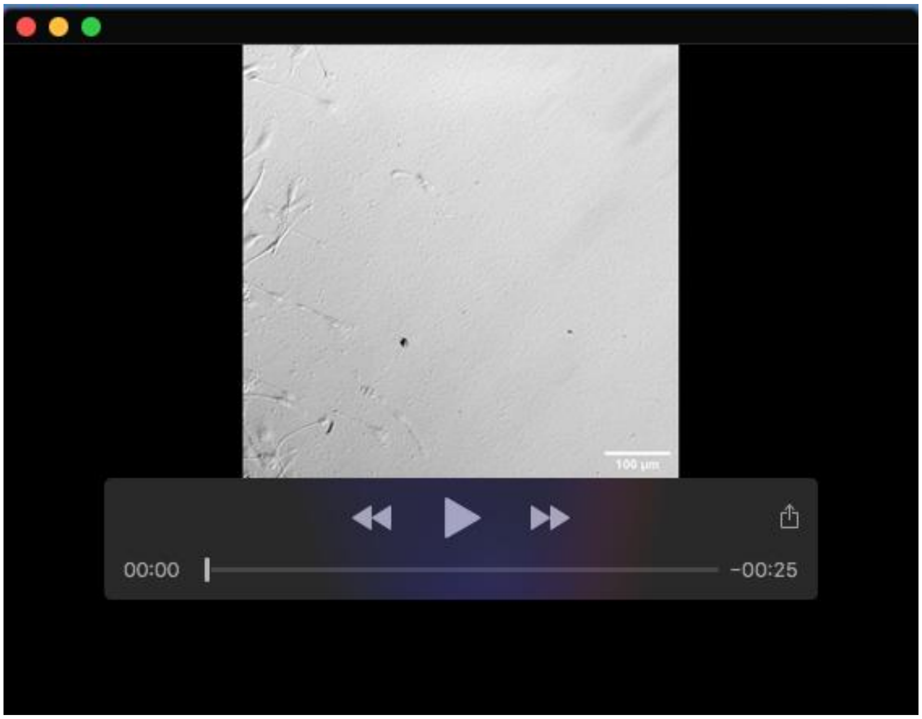

Movie 4.: Macrophages in Zone 2 (-) migrating on 10 ug/mL uniform fibronectin.

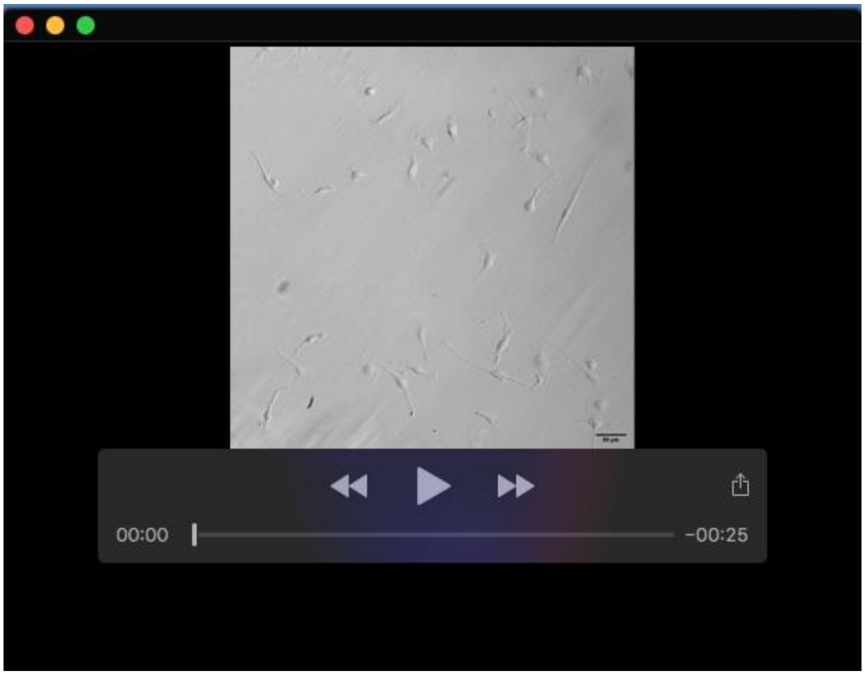

Movie 5. Macrophages in Zone 2 migrating on 10 ug/mL uniform fibronectin, after being directly injected under agarose.

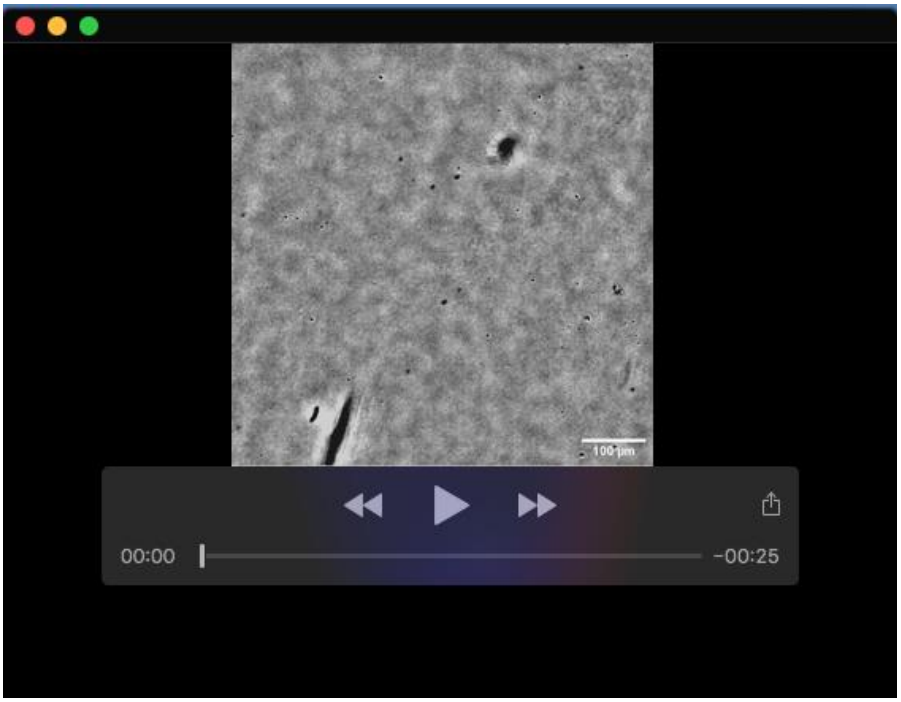

Movie 6.: Macrophages in Zone 2 (+) migrating on 250 µg/mL haptotactic gradient.

Biology Open • Supplementary information

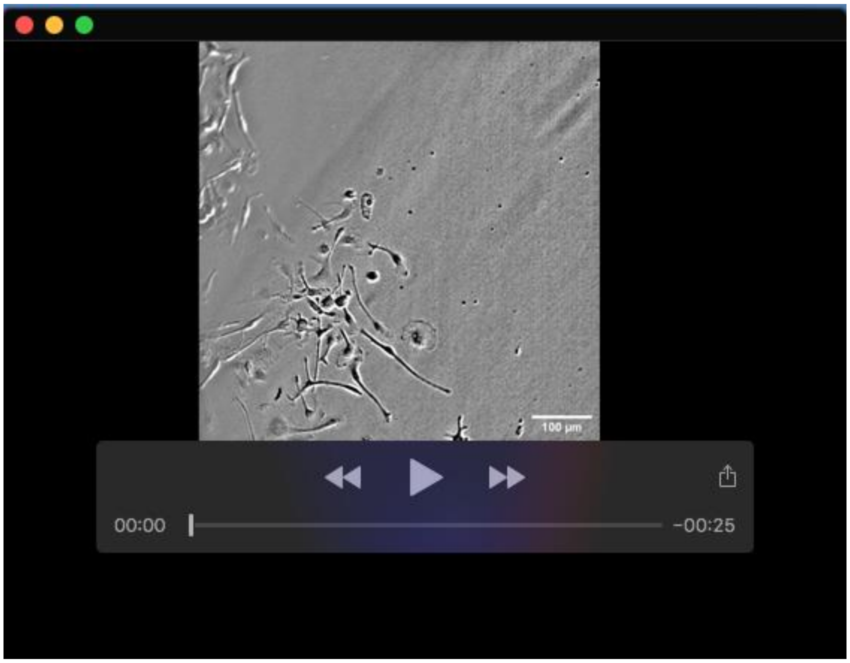

Movie 7. Macrophages in Zone 2 (-) migrating on 250 µg/mL haptotactic gradient.

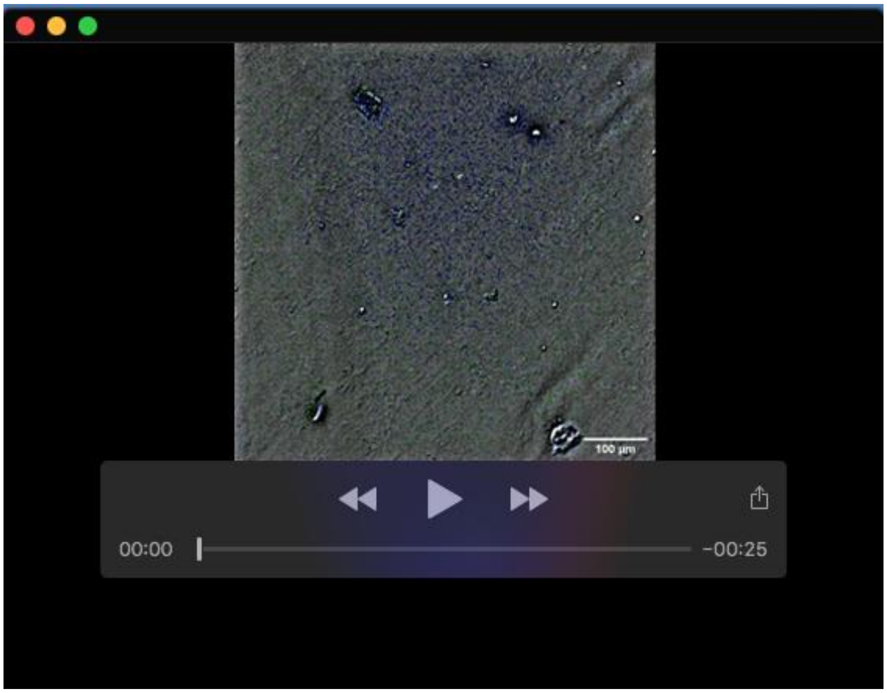

Movie 8.: Mixed population of WT and KO macrophages in Zone 2 (+) migrating on 250 µg/mL haptotactic gradient.

Biology Open • Supplementary information

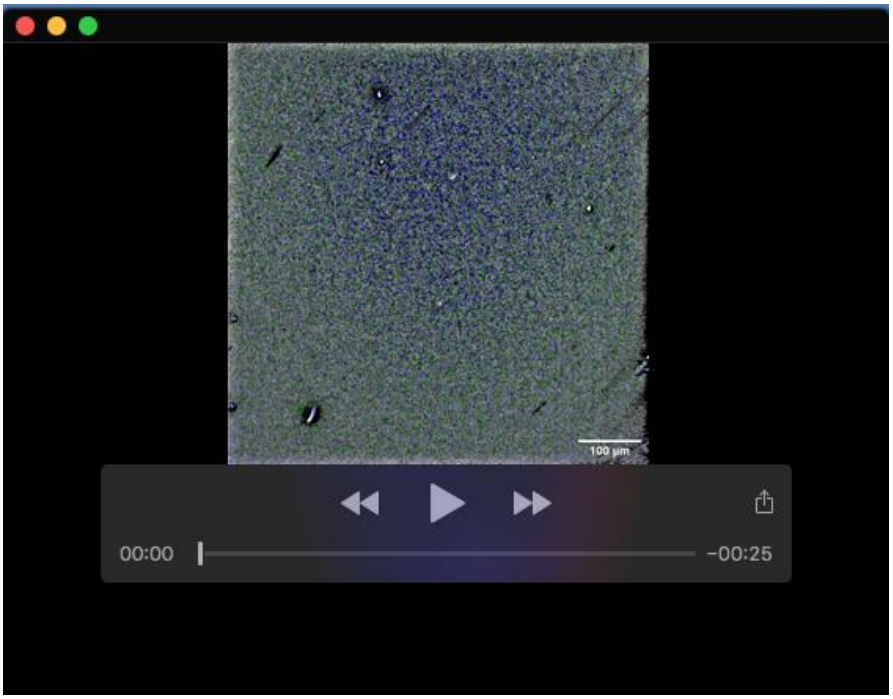

Movie 9. Mixed population of WT and KO macrophages in Zone 2 (-) migrating on 250 µg/mL haptotactic gradient.

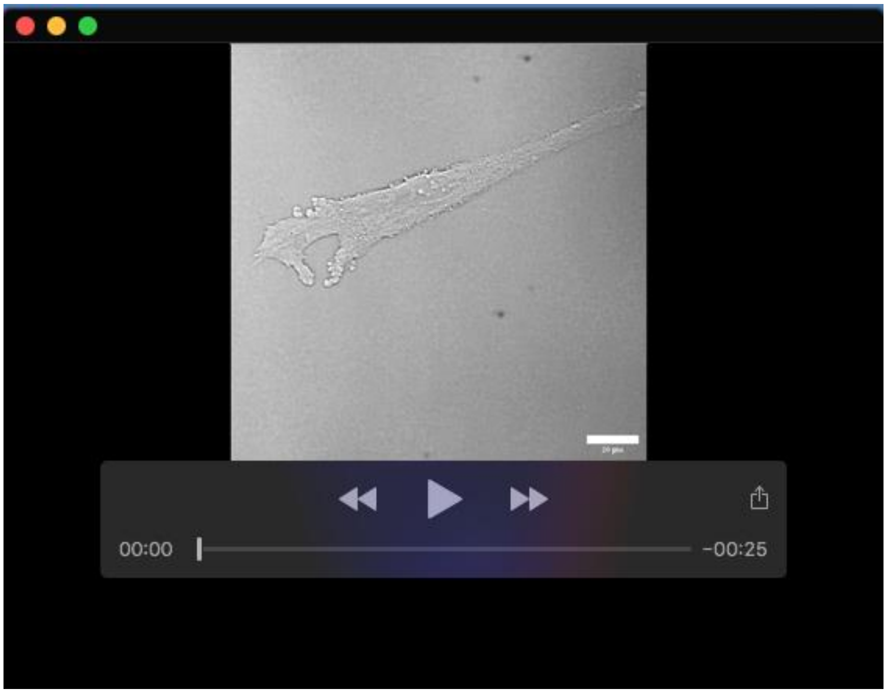

Movie 10. Example of bleb-producing *Arpc2*$^{-/-}$ macrophage migrating under agarose on 250 µg/mL haptotactic gradient, 40x magnification with 10s intervals.

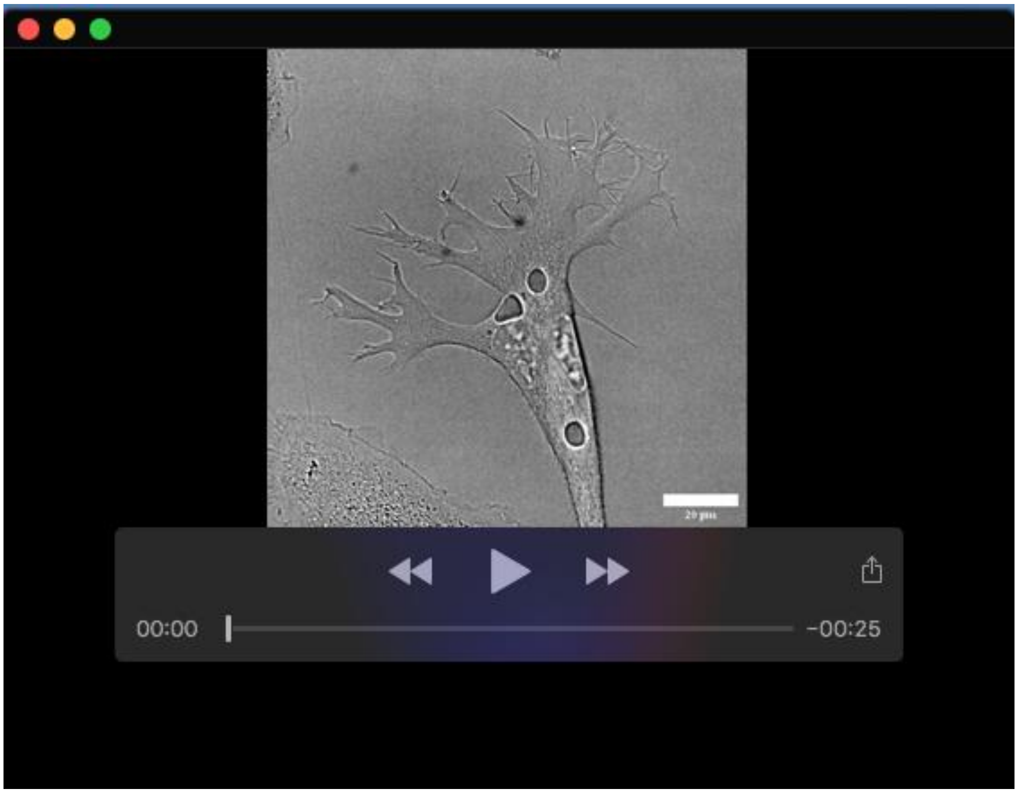

Movie 11. Example of filopodia-producing *Arpc2*$^{-/-}$ macrophage migrating under agarose on 250 μg/mL haptotactic gradient, 40x magnification with 10s intervals.
